# Residents’ Perspectives on Geriatric Psychiatry: A Tunisian Survey.

**DOI:** 10.1192/j.eurpsy.2023.1988

**Published:** 2023-07-19

**Authors:** M. Karoui, H. Nefzi, R. Kammoun, F. Ellouze

**Affiliations:** 1 Psychiatrie, Faculté de médecine de Tunis, Tunis, Tunisia; 2 Psychiatrie, Faculté de médecine de Tunis, Tunis

## Abstract

**Introduction:**

Despite the projected growth of the geriatric population, there is currently no clear treatment framework for these patients. This treatment requires specific training for psychiatrists in the field of geriatric psychiatry.

**Objectives:**

to evaluate the attitudes of psychiatric residents in Tunisia with regard to gerontopsychiatry.

**Methods:**

All psychiatry residents at Razi Hospital in Tunis in April 2022 were asked to complete an anonymous online survey with questions related to previous experience with the elderly, exposure to geriatric psychiatry patients during medical school, future career plans and interest in pursuing a geriatric psychiatry rotation, and factors involved in their decision.

**Results:**

55 of 72 (76%) residents responded, of whom 63 (n=34)% were in their second year of residency. 69% (n=38) of residents reported no exposure to geriatric psychiatry patients during medical school, but of those who had, 70% (n=24) had a positive experience. Only four residents (7%) reported considering a career in geriatric psychiatry. With respect to the geriatric psychiatry curriculum, all residents felt that changes were needed in geriatric psychiatry education and career path.

**Conclusions:**

Residents’ interest in further training in geriatric psychiatry is low. The most common reason is the perception of a poor prognosis for this patient population. Future studies are needed to develop strategies to increase interest in this field.

**Disclosure of Interest:**

None Declared

